# Supplementary material for: Peste des petits ruminants virus virulence is associated with an early inflammatory profile in the tonsils and cell cycle arrest in lymphoid tissue
Source: Microbiol Spectr. 2025 Feb 24;13(4):e03124-24. doi: 10.1128/spectrum.03124-24 (PMC11960121; doi:10.1128/spectrum.03124-24)
Supplement: Figure S1; Tables S1 and S2 — RT-qPCR for virus detection in organs and tables summarizing erosive-ulcerous and histological lesions. [file spectrum.03124-24-s0001.pdf]

S1 Fig. RT-qPCR on organs collected during the experiment.

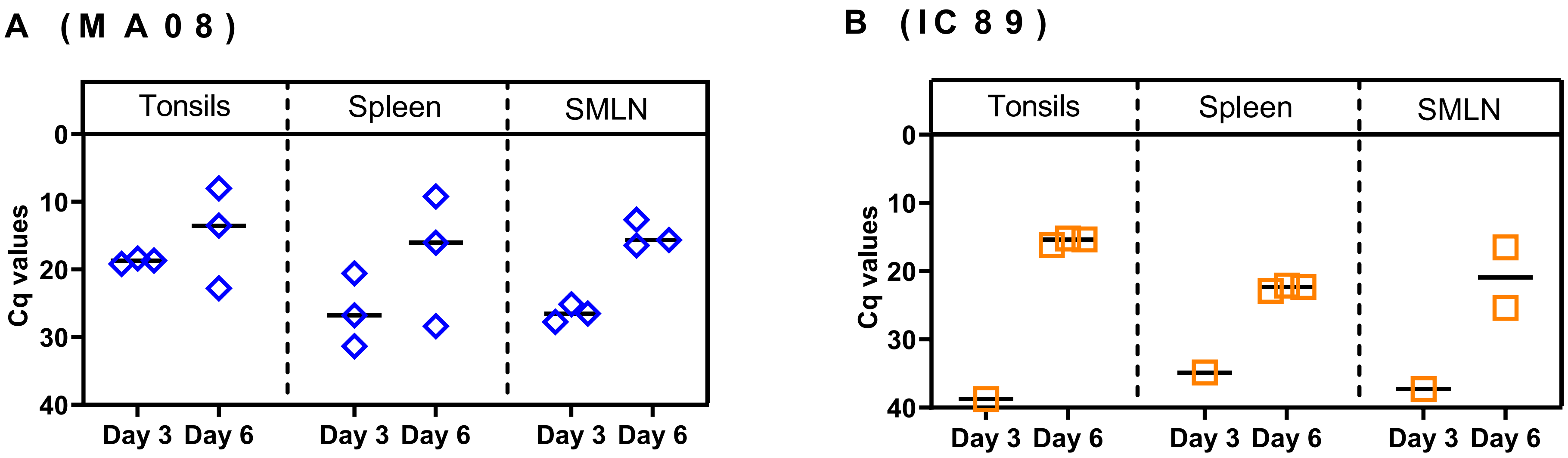

Supplementary table 1. Erosive-ulcerative lesions typical of PPRV-infection. Lesions were semi-quantitatively scored as follows: 0: absence; 1: focal or few multifocal; 2: Moderate and multifocal; 3: Multifocal to coalescing or diffuse, following the system reported by Eloiflin et al., 2022.

Group	Condition	Exp ID	Euthanasia day (dpi)	Erosive-Ulcerative lesions (0 to 3)			Accumulative scoring
				Palate	Lip	Tongie	
**85**	Mock CTRL	389	3	0	0	0	0
		836	3	0	0	0	0
		573	3	0	0	0	0
		540	6	0	0	0	0
		396	6	0	0	0	0
		522	6	0	0	0	0
**76**	Côte d'Ivoire 1989 (IC89)	526	3	0	0	0	0
		720	3	0	0	0	0
		588	3	0	0	0	0
		587	6	1	0	0	1
		390	6	1	1	0	2
		393	6	1	1	0	2
**86**	Morocco 2008 (MA08)	12	3	1	0	0	1
		560	3	1	0	0	1
		565	3	2	0	0	2
		681	6	2	2	1	5
		411	6	1	1	0	2
		394	6	1	0	1	2

Supplementary table 2. Histological PPRV-related lesions. The presence of lymphocytic depletion in tonsil and mesenteric lymph node was assessed semiquantitatively scored as described by Eloiflin et al., 2022.

Group	Condition	Exp ID	Euthanasia day (dpi)	Lymphocytic depletion (0-3)		Accumulative scoring (0-6)
				Tonsil	Mesenteric lymph node	
**85**	Mock CTRL	389	3	NA*	0	0
		836	3	NA	0	0
		573	3	NA	0	0
		540	6	0	0	0
		396	6	0	0	0
		522	6	0	0	0
**76**	Côte d'Ivoire 1989 (IC89)	526	3	NA	0	0
		720	3	NA	0	0
		588	3	NA	0	0
		587	6	0	0	0
		390	6	0	1	1
		393	6	1	0	1
**86**	Morocco 2008 (MA08)	12	3	0	0	0
		560	3	0	0	0
		565	3	0	0	0
		681	6	2	1	3
		411	6	1	1	2
		394	6	2	1	3
